# Distributed groundwater recharge potentials assessment based on GIS model and its dynamics in the crystalline rocks of South India

**DOI:** 10.1038/s41598-021-90898-w

**Published:** 2021-06-03

**Authors:** Fauzia, L. Surinaidu, Abdur Rahman, Shakeel Ahmed

**Affiliations:** 1 CSIR-National Geophysical Research Institute, Hyderabad, 500007 India; 2 Academy of Scientific and Innovative Research (AcSIR), Ghaziabad, 201002 India

**Keywords:** Climate sciences, Environmental sciences, Hydrology

## Abstract

Extensive change in land use, climate, and over-exploitation of groundwater has increased pressure on aquifers, especially in the case of crystalline rocks throughout the world. To support sustainability in groundwater management require proper understating of groundwater dynamics and recharge potential. GIS based studies have gained immense popularity in groundwater exploration in recent years because they are fast and provide recent information on the resource for future growth. Thus, the present study utilized a GIS-based Weighted Overlay Index (WOI) model to identify the potential recharge zones and to gain deep knowledge of groundwater dynamics. The in situ infiltration tests have been carried out, which is the key process in groundwater recharge and is neglected in many cases for WOI. In the WOI, ten thematic layers from the parameters influencing and involved in the recharge process are considered to identify potential recharge zones. The results suggested a significant underestimation of recharge potential without considering site-specific infiltration rates that one needs to be considered. The present WOI model considered in situ infiltration information and classified the entire area into four recharge zones, good, moderate, poor, and very poor. The final integrated map compared with the real-time field data like water level fluctuation and infiltration to analyse occurrence and quantification of recharge. The estimated average groundwater draft is 21.9 mcm, while annual renewable recharge is only 5.7 mcm that causing a continuous fall of the groundwater table. The study is useful in selecting regions with more focussed recharge studies and suggested the need of reducing groundwater demand by changing cropping patterns through a predictive decision support tool.

## Introduction

Groundwater resources have a significant role in marine and terrestrial ecosystems^1,2^. Groundwater supplies provide a reliable source for various purposes, including domestic, agricultural and industrial applications^3–7^. However, the recent trend of groundwater levels, especially in semi-arid regions of crystalline aquifers is depleting at alarming levels due to over-exploitation of groundwater than the recharge for various applications particularly for agriculture^8^. Groundwater recharge is a part of the hydrologic cycle that has a significant share in the water balance at the local, regional or global scale^9^. It assumes that the semi-arid areas are critical parts of the complete water balance of the earth’s sub-surface^10^. Surface water resource in semi-arid areas is limited; thus, to meet the water requirements for different uses, groundwater forms a reliable resource all over the world^11–13^. The recharge from precipitation to groundwater in semi-arid to arid conditions varies essentially in space, where the extreme climate of low and inconsistent precipitation and high annual temperature hampers the recharge^14^.

The efforts made to infiltrate precipitation water as aquifer storage and its quantification based on geospatial information systems make recharge efforts poorly successful over the globe^15,16^. Reliable estimation of recharge and its forecast remain challenging to numerous researchers and is an open-ended question due to understating on the origin and controls on infiltration and recharge mechanism particularly in the changing environment^14^. In the semi-arid areas, groundwater recharge takes place through various ways that can be incorporated; (1) direct recharge underneath rivers, streams and lakes, (2) focused recharge at the catchment margin, (3) aerially dispersed infiltration through the unsaturated zone^15,17^. Simply, recharge appears to increase in low topography zones with shallow-rooted vegetation and suitable soils that drain well^11,18,19^. Groundwater is a renewable natural resource; however, this vital life-sustaining resource recharge has been dramatically decreased over the last 4–5 decades due to different forms of anthropogenic activities and distorted innovations and technologies^20^. A better comprehension of groundwater recharge capacity is vital for water resource allocation and management for sustainable development. The increasing interest in GIS based studies in groundwater exploration in recent years owing to their pace and ability to provide first-hand information on the resource for future growth. The recent advancements in geospatial and digital image processing technologies have empowered researchers to better understand natural recharge processes by using a combination of semi-static information, for example, topography, geology, soil types, vegetation, etc.^14,21,22^.

Many researchers have applied geospatial techniques for the integration of different thematic layers, including climate, hydrogeological and hydrological information, to identify suitable recharge sites, for example, in south western Asia^12,21,23–28^, in Africa^29,30^ and in Arabian Peninsula^31,32^. None of the studies have considered site-specific soil infiltration rates which will have a major bearing on the estimation of recharge quantity in temporal and spatial scales. In the present study, we have attempted to overcome this limitation by integrating intensive insitu infiltration data and high-resolution soils with routine hydrology and hydrogeology data to understand its role in recharge dynamics and compare with existing integration methods to identify suitable recharge sites by taking Maheshwaram crystalline watershed located in South India. This research would aid in the decision-making process for successful agricultural planning and groundwater management.

### Description of the study area

**Figure 1:**
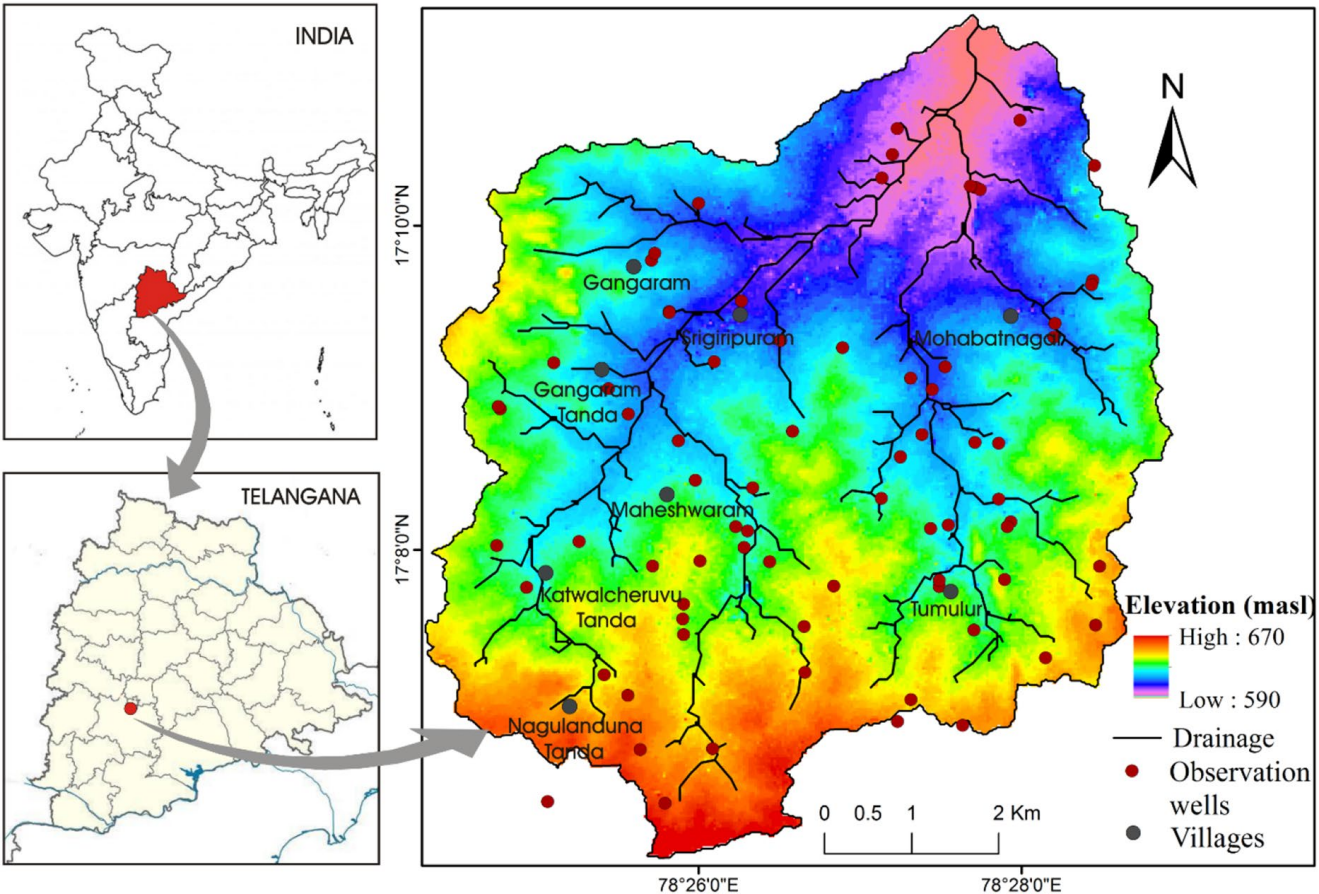
Location map of the study area showing elevation with the drainage system.

The Maheshwaram watershed is located 35 km south of Hyderabad in the state of Telangana that covers 53 km^2^ of the area. The area is characterized by moderate topography with an elevation range between 670 to 590 m above mean sea level (m, amsl) (Fig. 1) with a slope of about > 4 percent (Fig. 3a). There are no perennial streams in the area, and water flows in the streams during the rainy seasons only. The area encounters a semi-arid climate and is constrained by the regularity of the monsoon (monsoon period: June- October). Meteorological data from weather stations are obtained from local weather station and Customized Rainfall Information System (CRIS) (http://hydro.imd.gov.in) for the 12 years from 2008 to 2019 respectively. The mean annual rainfall is approximately 750 mm, over 90 percent of which occurs during the monsoon period. The average annual temperature is 26 °C; while the maximum daily temperature reaches 45 °C in the summer (March–May). The geology is generally homogeneous and composed of granites of the Archean age^33^. The study area is a representative catchment of Southern India regarding over-exploitation of its hard rock reservoir (more than 700 bore wells being in use), rural social economy (mainly based on conventional agriculture), its cropping patterns (rice field dominating), and agricultural practices. The main agricultural patterns in the area are rice, vegetables, and flowers, with some orchards of mangoes, guavas, and grapes^34^. The major source of water for irrigation in the area is groundwater.

#### Hydrological settings

Aquifers usually are formed in hard rock terrains by intense weathering for extended periods of time. Because of the occurrence of fractures, the aquifers are anisotropic and heterogeneous. Various mechanisms are invoked to describe the cause of the fractures, including cooling stress within the magma, subsequent tectonic movements^35^ or lithostatic mechanisms of decompression^36^. Nevertheless, some studies have shown that fracturing is the product of the weathering cycle^37,38^. The Maheshwaram watershed weathering profile is dominated by a multiphase weathering cycle caused by the Indian Peninsula's geodynamic history^38^. The weathering strata from the top downwards as follows:

- Red Soil (a thin 10–40 cm layer)
- Sandy regolith layer (1–3 m thick), locally covered by a lateritic layer (thickness < 50 cm)
- Laminated Saprolitic layer (10–15 m thick). The layer is distinguished by penetrating horizontal millimeter-spaced laminated structures and an irregular pattern of mostly sub-horizontal, as well as some partially filled nearly vertical fissures with clayey minerals retained. Both laminated saprolite layers and sandy regoliths have some granite corestones (preserved fresh rock).
- Fissured granitic layer (15–20 m) —In fissured fresh granite, some clayey minerals and the weathered granite generally filled the fissures partially.
- Bedrock—the unfissured granite.

Throughout the fissured granite and the laminated saprolite layer collectively forms the aquifer. Therefore, a two-tier structure, that is, weathered and fissured aquifers, coexists almost throughout the region. In general, when water-saturated, the laminated saprolite horizon is weakly transmissive with large volume, whereas the fissured layer is weakly capacitive, but strongly transmissive^40^. Besides, the lateral permeability in the study area is typically greater than that of the vertical one^41^.

In the study region the general groundwater flow direction is mainly regulated by topography, that is, from south to north. The water level was shallow before the inception of borewell irrigation in the 1970s and lay in semi-confined conditions in the saprolite region. Because groundwater has been over-exploited since the accessibility of borewells, the water level has decreased and the groundwater occurrence is now under unconfined conditions predominantly in the fissured horizon^40^. Borewell yields are moderate (negligible to 20 m^3^/hr) except those tap deep fissures in areas of tectonic origin, whereby yields are extremely high^41^. The groundwater water table depths range from 15 to 25 m in the study area and are isolated from surface water^41^. The main problem of the study area is unmanaged recharge and over-exploitation causes continuous depletion of water levels from the last decades. Our main objective of this study is to find out potential recharge zones that trigger managed aquifer recharge for the security of groundwater in the future.

## Materials and methods

### Data collection and processing

#### Infiltration

**Figure 2:**
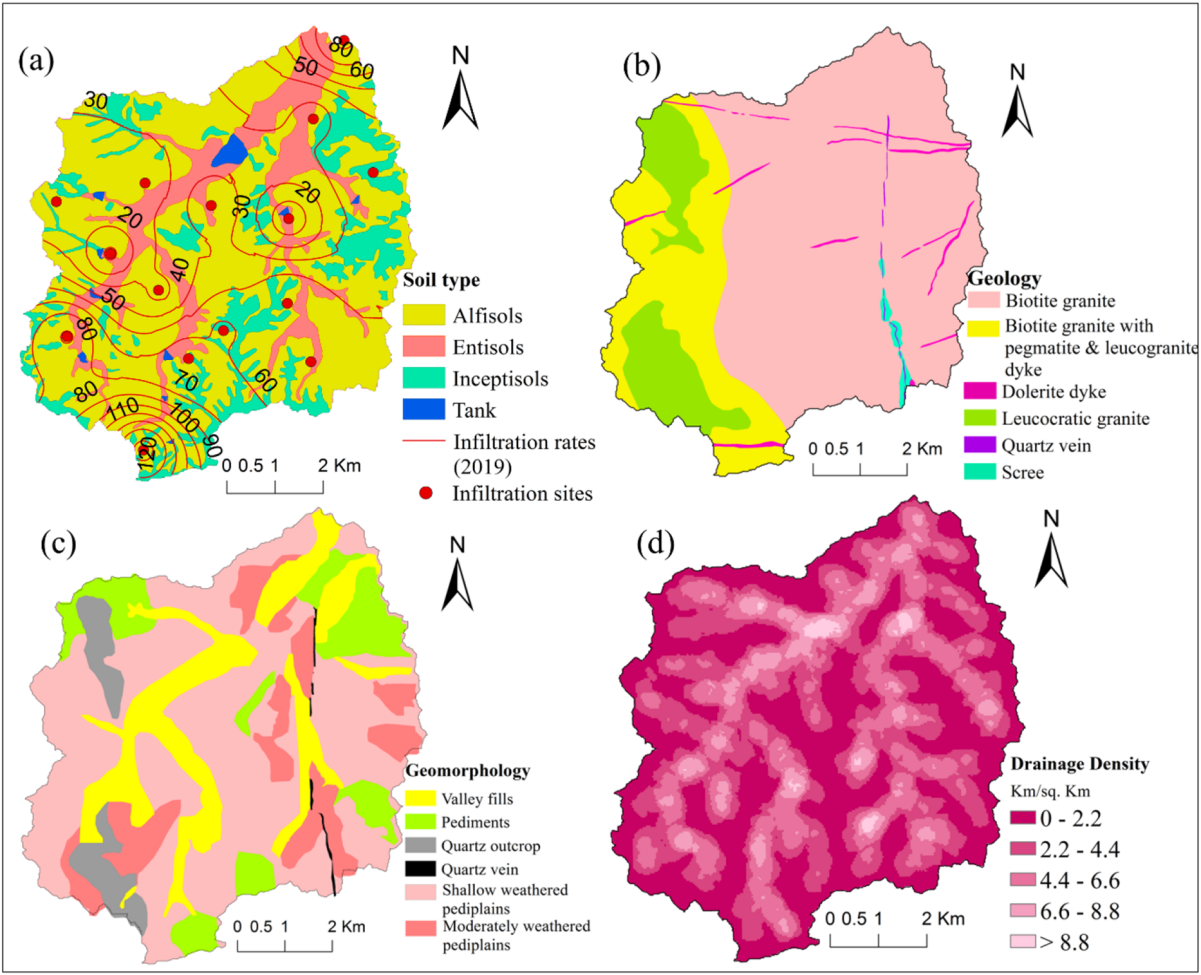
Map showing input thematic layers (**a**) soil with infiltration rates (mm/hr) and sites, (**b**) geology (**c**) geomorphology and (**d**) drainage density of Maheshwaram watershed, India. (ArcGIS Desktop. 10.3. ESRI, California, US. https://desktop.arcgis.com).

We have optimized the number of locations for carrying out infiltration tests based on soil type (Fig. 2a), and care has been taken to have at least one test for each soil type. Geomorphology, topography and land use are also considered while selecting the locations. A total of 15 infiltration locations were selected and distributed across the study area under different soil types and carried out during February 2019.

Double ring infiltrometer (Measurements as per ASTM D3385-03 standard test strategy)^43^ is used for soil infiltration tests in the study area. The diameters of the inner and outer rings of the double ring infiltrometer are 30 and 60 cm, respectively. They were set on the ground surface and safely fixed 10 cm into the ground. The outer ring helps to avoid any leakage in the lateral direction from the sides of the ring. From the inner ring, infiltration was recorded with the help of a stopwatch at 1 min interval for the first 6 min; every 2 min from 6 to 12 min; every 5 min from 15 to 50 min, every 10 min from 50 to 80 min, then every 20 min from 80 to 180 min and every 30 min from 180 to 240. The tests were carried out until there is no further infiltration of water. The estimation of infiltration rates was carried out by using Eq. (1):

1 $$Infiltration\,Rate\left( {Ir} \right) = \frac{a}{b} \times 60\,{\rm mm/hr}$$

2 $$Area\,of\,the\,ring \left( A \right) = 3.142 \times \left( {15} \right)^{2} {\rm cm^{2}}$$

where *a* is infiltration rate in mm, that estimated as the volume of the water added to the inner ring; *b* is the time interval (in minutes) between two successive readings.

#### Groundwater levels

The groundwater levels have been manually monitored two times a year (pre and post-monsoon season) using Water Level Indicator (WLI) from the year 2008 to 2019 through a dense network of observation wells, distributed across the area. The total depth of observation wells ranges from 32 m bgl to 60 m bgl and the location of these wells can be found in Fig. 1. Care was taken to prevent any interruption through pumping wells. The measurements were not taken in pumped wells and the rare instances of observable drawdown from the monitored wells owing to interference by adjacent pumping wells was no more than 10–20 cm, that is very less in comparison to water table fluctuations at the seasonal scale^33^*.*

### Thematic layers

Groundwater occurrence and movement are the functions of lithological, geomorphic, structural and hydrological parameters. Hence, a complete set of parameters is required to understand the groundwater system dynamics. In the present study, all thematic layers were extracted from secondary sources listed below, and the rationale for their integration is explained in the subsequent section in WOI. All maps in the paper were prepared using ArcGIS Desktop 10.3 (ESRI, California, US. https://desktop.arcgis.com).

#### Soils

Geology, physiography and climate describe soils and has a significant role in runoff and groundwater recharge^44^. Three kinds of soils are found in the investigation region, viz. Alfisols, entisols, inceptisols (Fig. 2a). For the study area soil, the mean particle-size fractions indicate the percent of clay, silt and sand in Alfisol are 19, 12 and 69%, and in Entisol 22, 21, and 56%, respectively^44^.

#### Geology

The geology of the area mostly covers Archean granites^45^. Biotite granite, quartz vein, dolerite dyke and leucocratic granite are the essential lithological units of the area (Fig. 2b). Quartz vein and dolerite dyke act as a barrier for the movement of groundwater^45^.

#### Geomorphology

The area is dominated by different depositional and erosional geomorphic characters, like pediments, pediplains, outcrops of rocks and valley fills (Fig. 2c). Area covered by shallow weathered pediplains is depicted about the flat landscape with a gentle gradient^45^. A fairly thick weathered material is a prevailing geomorphological part of the study area. Moderately weathered pediplains were found in different places of the area. Valley fills are commonly unconsolidated alluvial materials comprising of silt, sand, pebbles, and gravels accumulated along the base of the drainage valley.

#### Drainage density

The drainage was derived from 30 m resolution DEM using spatial analyst tools in ArcGIS. The key steps include sink filling, flow path recognition, flow accumulation calculation and definition of the stream^46^. The threshold value of 50 was selected for the drainage network extraction. Drainage density is characterized as the ratio of entire stream segment lengths in a basin to the area of the basin (Km/Km^2^). The range of drainage density varied between 0–11 km/km^2^. The drainage density of the investigation zone was set up by using a line density tool in ArcGIS software (Fig. 2d).

#### Slope

**Figure 3:**
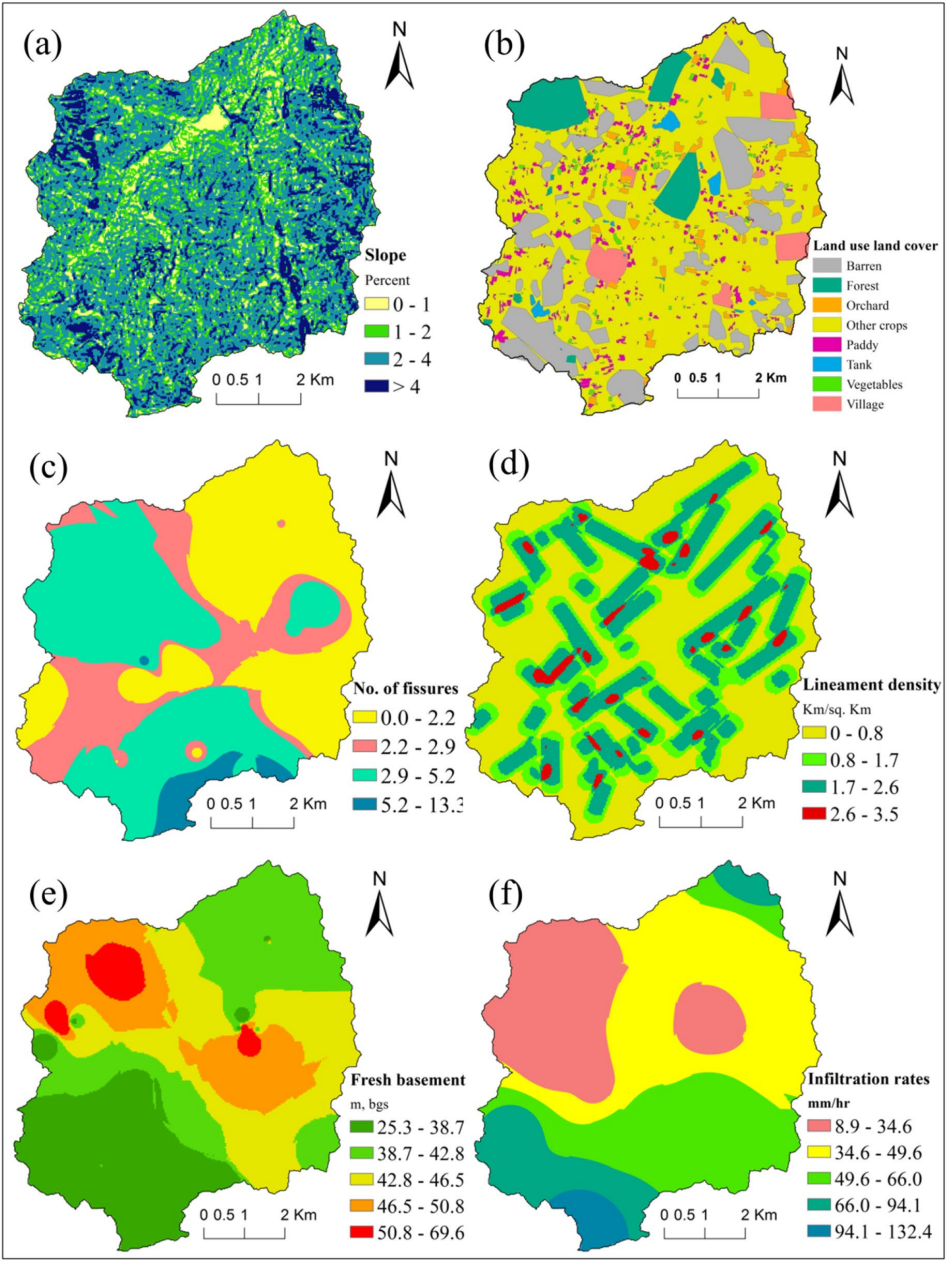
Map showing input thematic layers (**a**) slope, (**b**) Lulc, (**c**) fissures, (**d**) lineament density, (**e**) depth to basement and (**f**) infiltration rates of Maheshwaram watershed, India. Desktop. 10.3. ESRI, California, US. https://desktop.arcgis.com).

The watershed is dominantly a flat land^45^. The slope varies from 0 to more than 4 percent (Fig. 3a). Compared to the low-slope zone, a high slope will cause less infiltration and greater runoff^45^.

#### Land use and land cover

Land use and land cover (LULC) has an indispensable role in prospecting for groundwater^45^. Various LULC influence recharge rates, surface flow, and evapotranspiration^45^. LULC map has been prepared from LANDSAT 8 imagery with the help of unsupervised classification using ArcGIS and limited field visits. The derived land use maps in the Maheshwaram exhibits a range of categories include forest, orchard, paddy, villages, vegetables, barren land, tanks and other crops (Fig. 3b).

#### Fissure

A high-density horizontal fissure in the first few meters and the density of subhorizontal and subvertical fissures declining with depth are typically described by the fissured layer^35,39,40,42^. The fissured layer is believed to be the capacitive feature of the complex aquifer^37^. In the Maheshwaram watershed, fissured granite occupies 15–20 m, below the ground surface (m, bgs), the fissures are partly filled with a few clay minerals and weathered granite. Using flow meter measurements of 19 wells, the hydraulic conductivity and fracture density of conductive fissure zones were studied^39^, which established the depth position of hydraulically conductive fractures. The sum of all the fissures present in each well was taken separately to prepare the contour map for the recharge studies (Fig. 3c). Marechal et al. (2004) provided a complete explanation of the measurements and data interpretation method.

#### Lineament

Lineaments are characterized as naturally occurring linear or curvilinear surficial features^45^ that express subsurface geology and structural features like fault, fractures or joints^14^. Lineaments in the hard rock terrains represent regions and zones of fracturing and faulting that result in expanded secondary porosity and permeability and are acceptable pointers of groundwater^47^. Lineament map was taken from NRSC, Bhuvan and a density map was prepared using a line density tool in ArcGIS 10.3 (Fig. 3d). Lineament density is the total length of all the lineaments present in the unit area within the watershed. In the present study, the estimated density ranges from 0 – 3.5 km/km^2^.

#### Depth to basement

The depth of the basement is nothing but unfissured granitic bedrock which was derived by geophysical investigations and borewell lithologs for the study area^42^. Contour map for the fresh basement has been prepared using ArcGIS software (Fig. 3e) and the depth ranges from 25 to 69 m, below ground surface (m, bgs).

### Weighted Overlay Index

Weighted Overlay Analysis (WOA) is a method to produce integrated analysis by applying a similar range of values to input components^48^. The major processes in methodology include reclassifying each layer and integrating reclassified layers with suitable weights for the classification of groundwater recharge potentials. A total of 10 thematic layers are used in the study which governs the occurrence and movement of groundwater. The weights of these determining factors are based on their reactions to groundwater and expert opinion^20^. The total weights for all layers is equal to 100 percent^48^. A parameter with a high weight represents a layer with a large effect on groundwater recharge capacity, whereas a parameter with a low weight represents a layer with a slight impact^20^. In addition, the weights were assigned based on an analysis of previous research and field experience. The relative importance values of each parameter were allocated according to Saaty's scale (1–9)^20^. According to Saaty's relative importance scale, a value of 9 represents extreme importance, 8 indicates very, very high importance, 7 indicates very to extreme importance, 6 indicates strong plus, 5 indicates strong importance, 4 indicates moderate plus, 3 indicates moderate importance, 2 indicates poor, and 1 indicates equal importance.

Thematic layer sub-classes were re-classified using the natural breaks classification method in the GIS platform before being assigned weight. On a scale of 0 to 9, the sub-classes of each thematic layer rank were assigned according to their relative impact on groundwater occurrence^20^. The allocated rank and weights of thematic layers are shown in Table 1.

**Table 1:** Thematic layers used in weighted overlay analysis, their weights and class rank assigned for the preparation of potential recharge zones (Litholog and soil data is provided by Indo-French Centre of Groundwater Research, CSIR-NGRI).

Layers	Data sources	Weights (without infiltration) (Fig. 5a)	Weights (with infiltration) (Fig. 5b)	No. of classes	Class description	Ranks
LULC	Knowledge-based supervised classification technique and maximum likelihood classifier (generated in the present study)	12	9	8	Barren	1
					Village	2
					Vegetables	6
					Other crops	5
					Paddy	7
					Orchard	7
					Forest	8
					Tanks	9
Geology	Dewandel et al. (2006) (https://www.sciencedirect.com/science/article/pii/S0022169406001673#fig2)	7	6	6	Biotite granite	4
					Biotite granite with pegmatite and dyke	5
					Scree	4
					Leucogranite	2
					Quartz vein	6
					Dolerite dyke	7
Geomorphology	Modified from Rashid et al. (2012) https://link.springer.com/article/10.1007/s10661-011-2305-2/figures/5)	8	7	6	Shallow weathered pediplains	4
					Moderately weathered pediplain	5
					Quartz outcrop	1
					Quartz vein	6
					Pediments	3
					Valley fills	9
Soil		20	12	4	Inceptisols	5
					Alfisols	8
					Entisols	7
					Tanks	9
Lineament density (Km/sq. Km)	NRSC, Bhuvan (https://bhuvan-app1.nrsc.gov.in/thematic/thematic/index.php)	20	14	4	0–0.8	4
					0.8–1.7	6
					1.7–2.6	8
					2.6–3.5	9
Drainage density (Km/sq. Km)	SRTM DEM (30 m resolution)	8	8	5	0–2.2	8
					2.2–4.4	7
					4.4–6.6	6
					6.6–8.8	5
					> 8.8	4
Slope (in percent rise)	SRTM DEM (30 m resolution)	15	14	4	0–1.0	8
					1.0–2.0	6
					2.0–4.0	4
					> 4	2
Total number of fissures		10	10	4	0–2.2	6
					2.2–3.9	7
					3.9–5.2	8
					> 5.2	9
Depth of fresh basement rock (m, bgs)			10	5	25.3–38.7	4
					38.7–42.8	6
					42.8–46.5	7
					46.5–50.8	8
					50.8–69.6	9
Infiltration rate (mm/hr)	Insitu field data		10	5	8.9–34.6	4
					34.6–49.6	6
					49.6–66.0	7
					66.0–94.1	8
					94.1–132. 4	9

Thematic maps mentioned above were reclassified and georeferenced to a standard point of reference in the Universal Transverse Mercator (UTM) plane coordinate system, followed by the weights assigned as per their possible impact on groundwater recharge using the Weighted Overlay Index method^49,50^.

The weights are given to respective layers to give relative significance to recharge^14,20,26^. Each thematic layer reclassified to low, moderate, and high from the lowest to the highest recharge potential dependent on natural gaps in the data. All thematic layers have been integrated with the weighted overlay analysis method in GIS platform using Eq. (3) to generate groundwater potential zone map of the basin^20^.

3 $$GWPZ = \mathop \sum \limits_{i}^{n} \left( {X_{A} \times Y_{B} } \right)$$

where GWPZ = Groundwater Potential Zone, *X*- denote the weight of the thematic layers; *Y* – denote rank of the thematic layers (sub class). The term ‘A’ (A = 1, 2, 3,…, X) addresses the thematic map and ‘B’ (B = 1, 2, 3,…, Y) addresses the classes of thematic map. ‘i’ represent first thematic layer and ‘n’ represent the number of layers.

**Figure 4:**
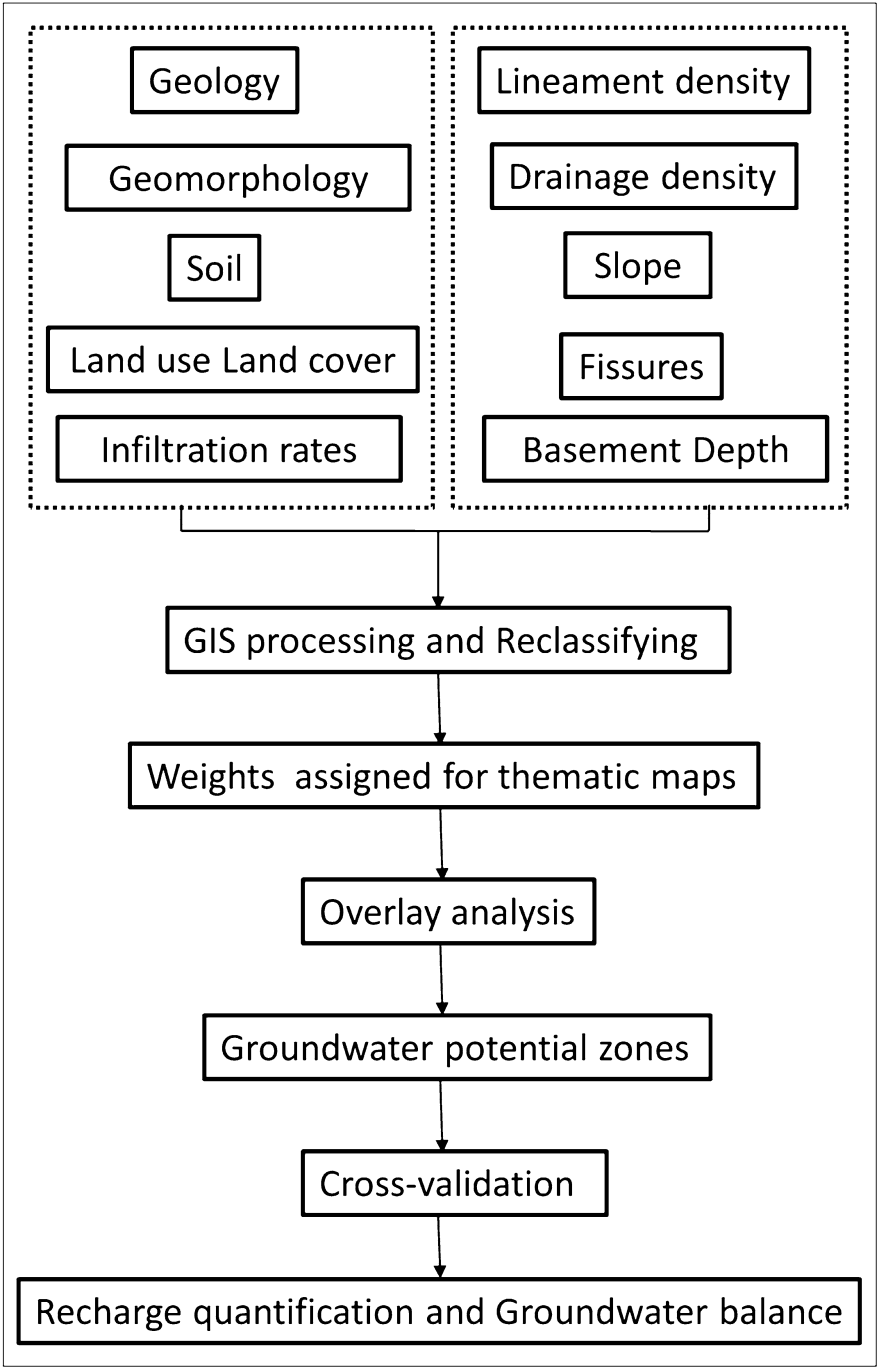
Flow chart showing WOI methodology adopted in the study area for the evaluation of potential groundwater recharge zones.

Figure 4 shows the methodology adopted in the current study for the integration of different layers followed by overlay analysis, cross-validation and recharge quantification. Weights based on priority for groundwater recharge are presented in Table 1. Depending on the knowledge-based hierarchy, the different classes of each theme were allocated from 1 to 9 ranking. The overall weights of the final integrated map were obtained as product or sum of weights allocated to different layers as per their suitability. The final map was categorized into four distinct groundwater recharge zones that are good, moderate, poor, and very poor. The technical guidelines set by the National Remote Sensing Agency^51,52^ are used for the preparation of recharge map.

### Estimation of groundwater recharge and draft

The recharge is measured by the water table fluctuation method using the following formula^53^:

4 $$Recharge = Geographical\,area \times Water\,table\,fluctuation \left( {difference\,between\,post\,to\,premonsoon} \right) \times Specific\,yield$$

Specific yield (Sy) values were considered for the study is taken from the Ground Water Estimation Committee’s recommended values^54^.

The percentage of precipitation converted into groundwater recharge is shown in Table 3. Owing to scanty rainfall and lack of surface water sources, the study area relies mainly on groundwater for its irrigation. More than 70 percent of available groundwater resources are used by most of the administrative units (mandals).

In the current study, the draft of groundwater has been estimated based on detailed land-use statistics that were provided for the years 2008, 2014 and 2019. The detailed data is provided in Table 3. In this method, the groundwater draft has been calculated by multiplying various irrigated crops area (cultivated using groundwater) with the crop water requirement for each crop^53,55^. The method is popularly named as irrigated area statistic method, initially recommended by GEC 2009^53^.

The following formula has estimated the total groundwater drafts for irrigation^53^:

5 $$Total\,draft = Irrigated\,area \times Crop\,water\,requirement$$

For paddy, the crop water requirement taken is 0.95 m for monsoon, and horticulture and irrigated dry it is 0.6 m and 0.45 m for non-monsoon, respectively (after removing 50 percent of rainfall, i.e., 0.25 m)^53^. In the area, paddy (rice), vegetables (tomatoes, ladies' fingers (okra), brinjals, chilies, etc.), fruits (mangoes, guava, and grapes) and flowers are considered for the estimation of groundwater draft (Fig. 3b).

## Results and discussion

### Infiltration

Infiltration is one of the most critical hydrological processes that drive the recharge to the vadose zone and the groundwater. The fundamental way of evaluating groundwater resources is by use of Infiltration rates^56^. The infiltration rates vary widely across the area from 132.5 to 8.9 mm/hr, such a wide variation could be explained by the nature of the soil, vegetation, geology, slope, etc. The percentage of sand, silt, and clay in a soil is the most significant inherent factor that affects infiltration. Water infiltration is faster through larger pores such as in sandy soil than through smaller pores like clayey soil. Low infiltrations may be expected if the clay is compacted and lacks structure or aggregation^57^. The spatial distribution of infiltration rates are provided in Fig. 2a and data is provided in Table S1 as a supplementary material, which shows that it is highest in the southernmost and northernmost part of the region whereas it is lowest in the central and western part (Fig. 3f). Less infiltration rate at some places is primarily due to more clayey and silt in soils. Entisols are showing lower infiltration rates, which is possible because their soil profile contains more silt as compared to alfisols. Whereas alfisol soil has shown comparatively higher infiltration rates than entisol and inceptisol. Siltation is one of the most important reasons for low recharge. It acts as a barrier and does not allow the water to infiltrate beneath^45^. When it comes to geology, it is obvious that hard and more resistant rocks do not allow the water to infiltrate, moreover, weathered and less resistant rocks will promote more recharge in the region. Vegetation is another reason that promotes infiltration by increasing water penetration time into the soil. The higher the vegetation, the more will be the infiltration, and lower vegetation will often lead to barren land. In the study area, the highest infiltration rates are observed in cropland areas. Moreover, drainage density also impact the infiltration capacity and can be noticed lower rates of infiltration at high drainage density regions whereas low drainage density areas show higher infiltration rates.

### Integration of thematic layers and their significance to groundwater recharge

Thematic layers (geology, soil, geomorphology, lineament density, LULC, drainage density, fissures, and slope) were reclassified, followed by the weight assigned as per their relative impact on groundwater recharge (Table 1). Geology and geomorphology of a region are very significant characteristics in assessing an area's groundwater recharge zones^58^. High weights are assigned for quartz vein and dolerite dyke that act as a barrier for the movement of groundwater. Dolerite dyke are distributed in different parts of the area and oriented in E-W and SE-NW directions. Whereas, quartz vein following the N–S trend in the south-western part (Fig. 2b). A large part of the area is geologically occupied by biotite granite and biotite granite with pegmatite dyke, assigned higher ranks as they are more deeply weathered than leucocratic granite. Similarly, the high weight assigned for valley fills as they are mostly unconsolidated sediments that promote high recharge in the area. Valley fills are found in the middle parts following the drainage network of the study area (Fig. 2c). The region is primarily secured by coarse material with great vegetation spread. While the area covered by shallow and moderately weathered pediplains assigned as moderate, pediments are assigned as low to moderate as pediments comprise the lower weathered zone. A smooth and level buried pediment surface comprises of shallow overburden of weathered material and is found mainly at the south western part while in the northern part of the region there are a few scattered patches. The low weight assigned for quartz outcrop, found in the western and south western part of the investigation area. Quartz is very resistant to weathering and does not support recharge. Negligible vegetation is found around rocky area. The water-holding capacity of a region relies on the types of soil as well as their permeability. The first step of transmission and infiltration of surface water to subsurface water is a function of texture and soil type^59^. A higher rank assigned to alfisol because it has less silt than entisol soil that offers more percolation, and hence recharging (Fig. 2a). Drainage density and slope are the crucial parameters that control runoff and the infiltration of the area. Drainage density is a reverse function of permeability^45^. The lesser the permeability of a rock means, the lesser the infiltration of precipitation, which will then flow as surface runoff. It offers a well-developed and well drainage system. Higher drainage density shows low infiltration and consequently acts as poor groundwater recharge zones contrasted with low drainage density suggesting a reverse connection between the two. In the present study, the comparison of infiltration test results with drainage density map revealed that higher infiltration rates are prevailed at low drainage density region whereas the opposite is observed at higher density regions (Fig. S1). However, the slope is an important characteristic of terrain that expresses the steepness of the ground surface. The slope is compatible with the shallow groundwater hydraulic gradient and controls water runoff speed and infiltration rate^60^. Larger slopes yield less recharge because, during rainfall, the water obtained from the rainfall moves immediately down the steep slope. Thus, the saturated zone does not have enough residence time to infiltrate and recharge^20^. The drainage density and slope map of the Maheshwaram basin are shown in Fig. 2d and 3a, respectively. Drainage density of the area was reclassified and categorized as very low (0–2.2), low (2.2–4.4), high (4.4–6.6); (6.6–8.8), very high (> 8.8). The high drainage density assigned lower weights, whereas low drainage density assigned higher ranks. The presence of strongly resistant and permeable rock is indicated by the poor drainage course network, while a higher drainage course shows extremely weak and impermeable rocks^61^. Slope values are reclassified and graded into five groups for flat, gentle and steep slopes, such as flat (0–1), gentle (1–2), steep (2–4), and very steep (> 4). High weights are given for flat and gentle slopes, whereas lower weights for steeper slopes. Groundwater recharge is high close to lineament zones as lineaments have a significant role in the recharge of groundwater in hard rock areas^62^. Lineament density (Fig. 3d) are reclassified and identified as low (0–0.8), moderate (0.8–1.7), high (1.7–2.6), very high (2.6–3.5). High lineament density assigned higher weights, as it allows water to percolate and is a good indicator of recharge zones. While the lower weights assigned to lower density accordingly. Fractures and faults play a very important role in groundwater replenishment as the zones of fracturing and faulting result in expanded secondary porosity and permeability and are acceptable pointers of groundwater recharge^47^. According to the available data, the sum of the fissures presents in each well is taken for the recharge studies. The fissure map for the Maheshwaram basin is shown in Fig. 3c. For the present study, fissures are reclassified and categorized as low (0–2.2), moderate (2.2–2.9), high (2.9–5.2), and very high (> 5.5). More number of fissures are assigned more weights, and fewer fissures are assigned less weights. Similarly, infiltration rates and fresh basement depth (Fig. 3e and f) are reclassified and categorized into five classes. Infiltration rates as very low (8.9–34.6), low (34.6–49.6) moderate (49.6–66.0), high (66.0–94.1), very high (94.1–132.4) whereas very shallow (25.3–38.7), shallow (38.7–42.8), moderate (42.8–46.5), deep (46.5–50.8), very deep (50.8–69.6) for basement depth. Infiltration is a primary process in groundwater recharge. The method of infiltration suggests that a part of precipitation reaches the water table^56^. Infiltration rate is directly proportional to vegetation density, for example, the runoff will be less and infiltration will be more if the area is covered by heavy forest. The runoff yield is expanded from the area steadily from forest spread, grassland, agricultural land, barren land and urbanized developed land^59^. Water bodies are an important source of direct and continuous recharge. Water bodies and forests are allocated the most elevated position for groundwater recharge. The paddy fields, orchids and crop plantation with great vegetation spread advances the rate of infiltration and prevents excess runoff and in this way are allocated high weights for groundwater recharge. Rocky areas, barren lands and villages are given low weightage since the water penetration rate is poor. The distinguished groups of land use are mainly paddy, orchids, different crops (like maize, vegetables, cotton, sunflower), forest, rocky outcrops, barren land and tanks (Fig. 3b).

### Potential groundwater recharge areas

**Figure 5:**
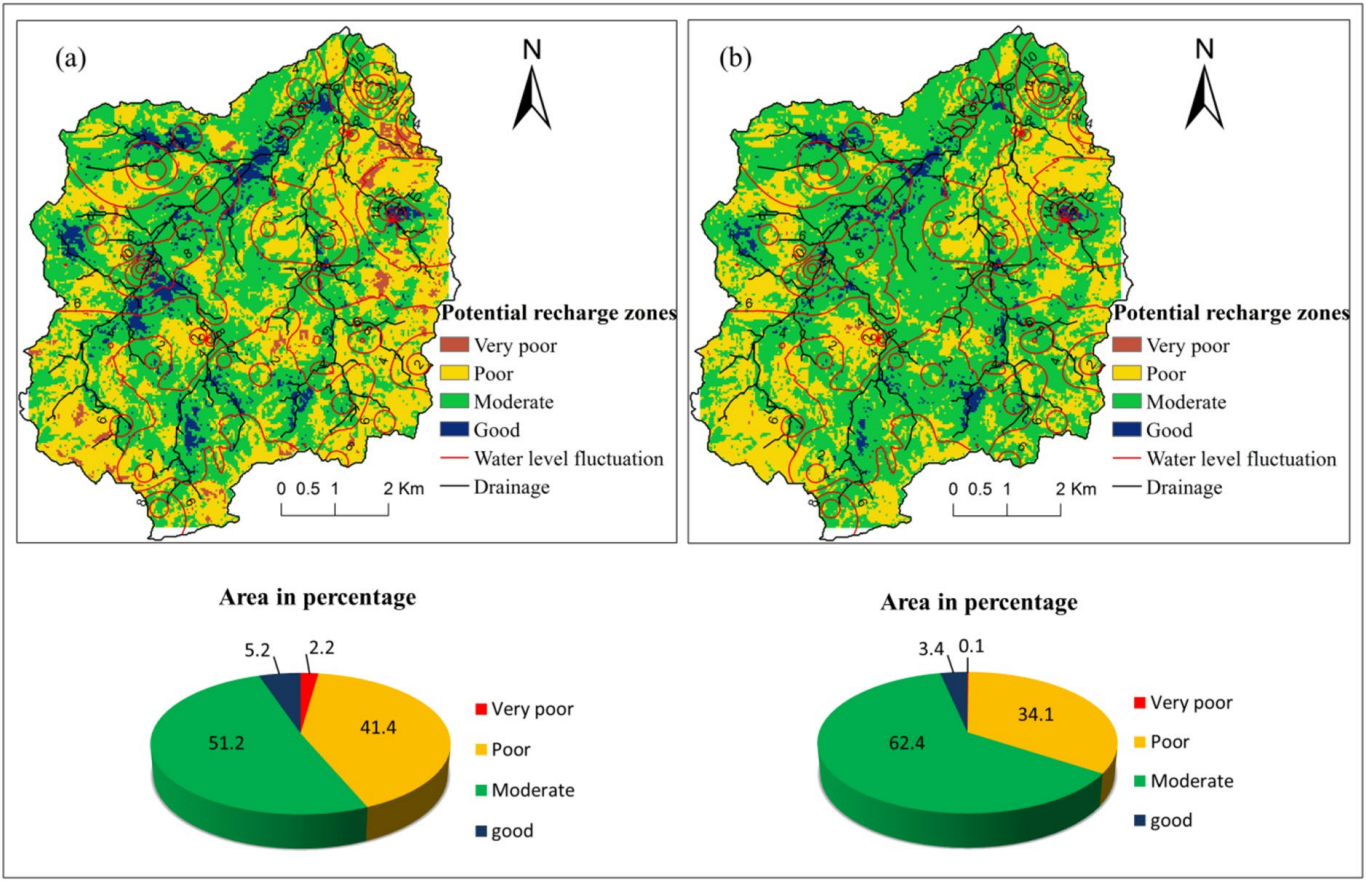
Potential groundwater recharge zones (**a**) without infiltration rates and basement depth (**b**) with infiltration rates and basement depth. Desktop. 10.3. ESRI, California, US. https://desktop.arcgis.com).

In the present study, two different types of groundwater recharge potential maps were prepared using WOA. The first one (Fig. 5a) is prepared using routine hydrogeological parameters listed in Table 1 that most of the WOA analysis methods use. The second one is considering site-specific detailed soil infiltration rates and basement depths which will provide a detailed recharge estimation to support local level groundwater management. The resulting maps have been classified as good, moderate, poor, and very poor groundwater recharge zones (Fig. 5). The area of potential recharge zone has been calculated as 2.2, 41.4, 51.2 and 5.2% for the very poor, poor, moderate and good, respectively for the first case (Fig. 5a), and in the second case, it is 0.1, 34.1, 62.4 and 3.4% for very poor, poor, moderate and good, respectively (Fig. 5b). The comparison of potential groundwater maps in the two cases indicated that underestimation of about 11% of potential groundwater recharge areas in the absence of site-specific soil infiltration rates and basement depths. The potential recharge zone scenario in Fig. 5b had changed when infiltration rates and basement depth were considered. Poor and very poor recharge zones decrease while the moderate recharge zones impressively increased as compared to Fig. 5a. Figure 5b indicates the potential recharge zones are highly influenced by basement depth and infiltration rate of the area.

As seen from Fig. 5, good groundwater recharge zones often occur in the middle of the region while a few patches are found in the eastern, north-western and southern part. High groundwater recharge areas are typically limited to the valley and low drainage density areas that have good infiltration potential. The moderate recharge zones are usually distributed all over the area, mainly in the shallow weathered pediplains of biotite granite and biotite granite with pegmatite and dyke and low lineament density. The poor and very poor recharge zones occur primarily in highlands to lowlands, but very poor recharge zones are relatively less in the middle parts. The poor and very poor recharge zones found in the rocky outcrop, high drainage density, steep slope, less lineament density, less no. of fissures and barren land regions.

### Correlation of potential groundwater recharge zones with water level fluctuation and rainfall

**Figure 6:**
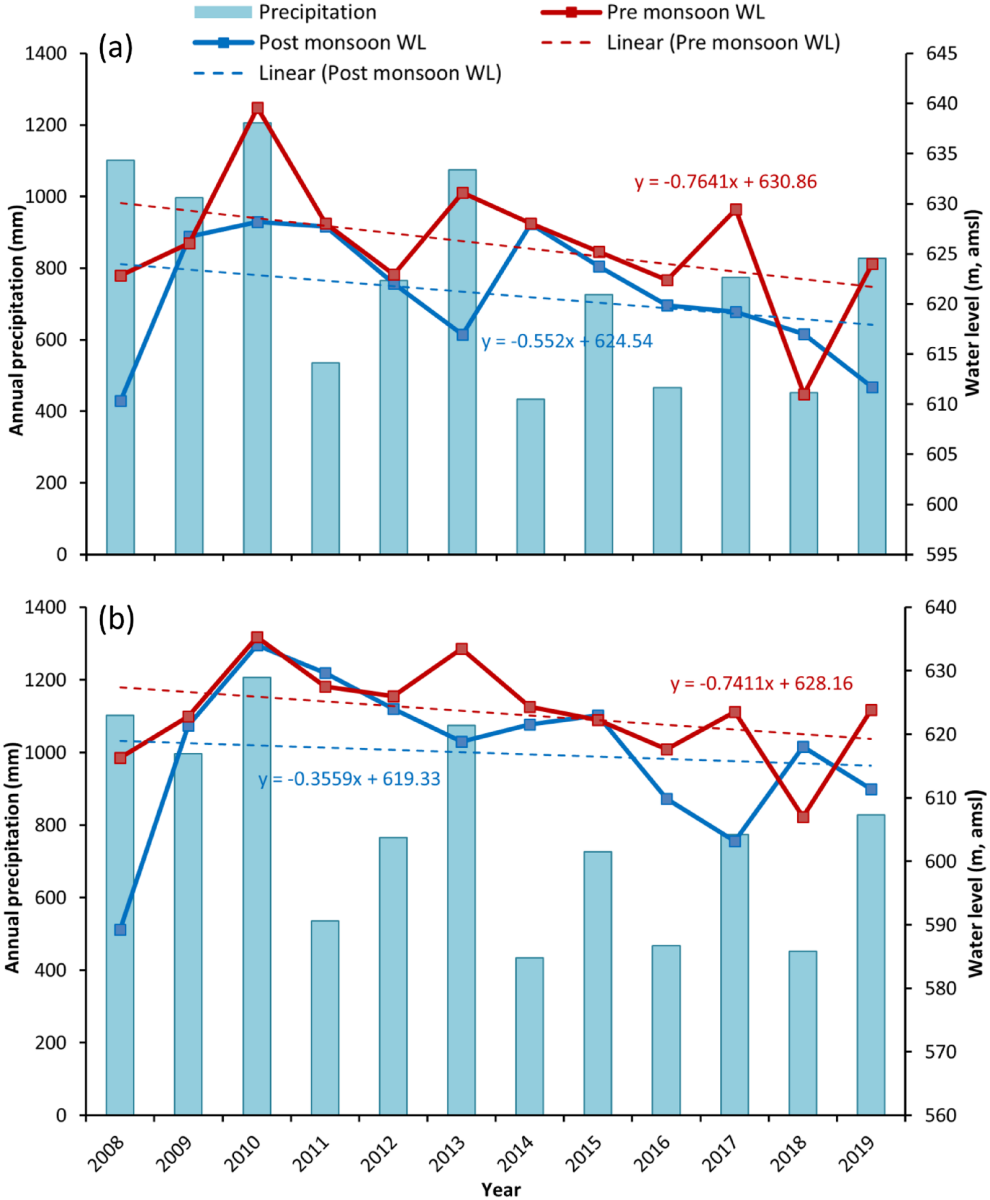
Graph showing the temporal variation of water level fluctuation (meter, above mean sea level) in the areas of (**a**) low recharge zone and (**b**) high recharge zone.

To check the validity of potential recharge maps generated from the WOA method, groundwater recharge zones delineated in this analysis are further crossed-checked with water level fluctuations from the year 2008 to 2019. Water level data have been collected twice a year during the pre and post monsoon period. The observations revealed that the aquifer exhibits significant seasonal water-level fluctuations due to monsoon recharge through a relatively thick unsaturated zone. The careful observations revealed that water level fluctuation is higher in the northernmost that is 12 to 16 m, eastern and western parts of the study area it is 10 m where WOA demarcated the zone as good to moderately recharge area (Fig. 5a and b). Whereas, poor recharge zones are showing less water level fluctuation can be seen in Fig. 5a and b. The recharge mostly occurs during the monsoon rainfall that occurs for 4 months, July to October, and the rest of the period there is only pumping. A few light spells of rainfall do not contribute to the recharge. Thus, the hydrological cycle can be clearly divided into two distinct seasons. Measuring and plotting water levels will certainly provide some minor details but do not change the trend. The average of pre and post-monsoon water levels from all the wells falling in low and high recharge zones shows that observed groundwater elevations vary between 592 and 640 m amsl in the area. In the area, groundwater fluctuations are more consistent with rainfall and show a steady declining trend in both pre and post monsoon during the observed period between the year 2008 to 2019 (Fig. 6). The wells located in potentially very low groundwater recharge areas are observed to show fewer fluctuations in water level from pre to post-monsoon, which eventually results in low recharge.

On the other hand, high groundwater recharge zones showing more pre and post-monsoon water level fluctuations that may result from more recharge. It is observed that higher fluctuations are at paddy fields and near areas, which may be due to high pumping inducing more recharge (Fig. S2). It is also observed that wells near streams have shown high post monsoon groundwater level fluctuations that indicate critical surface–groundwater interactions.

### Groundwater management for agriculture sustainability

The estimated recharge rates vary widely within land use land cover settings. It has been noted that most of the paddy and forested areas with some orchards, vegetables and double crops are under the moderate potential recharge zones, whereas some paddy and vegetables and a small part of the forest is coming under good recharge zones for the year 2008, 2014 and 2019 (Table 2). At the same time, mixed plantations and some paddy and orchards have been practicing in the poor recharge zone. The differences in land use land cover thus contribute to the wide variations in recharging processes. The relationship between land use land cover settings and groundwater recharge apparent in the current study allows for a better evaluation of the effect of future land-use changes on groundwater quantity.

**Table 2:** Land use land cover in different potential recharge zones.

Recharge zones	Year			Average recharge mcm
	2008	2014	2019	
Good (1.75 Sq. km)	Some paddy, double crops	Paddy, vegetables	Small part of forest, vegetables	0.216
Moderate (32.93 sq. Km)	Some paddy, orchards, vegetables, double crops, forest	Mostly paddy, forest, vegetables	Forest, vegetables, paddy, orchards, other crops	3.882
Poor (17.71 sq. Km)	Paddy, orchards, vegetable, mixed plantation, build-up, barren, some area of forest	Some paddy and forest, build-up	Some forest, mostly barren, some paddy and orchards and other crops, build-up	1.619
Very poor (0.06 sq. Km)	Barren, build-up	Build-up	Mostly barren	0.002

### Groundwater draft and recharge

**Figure 7:**
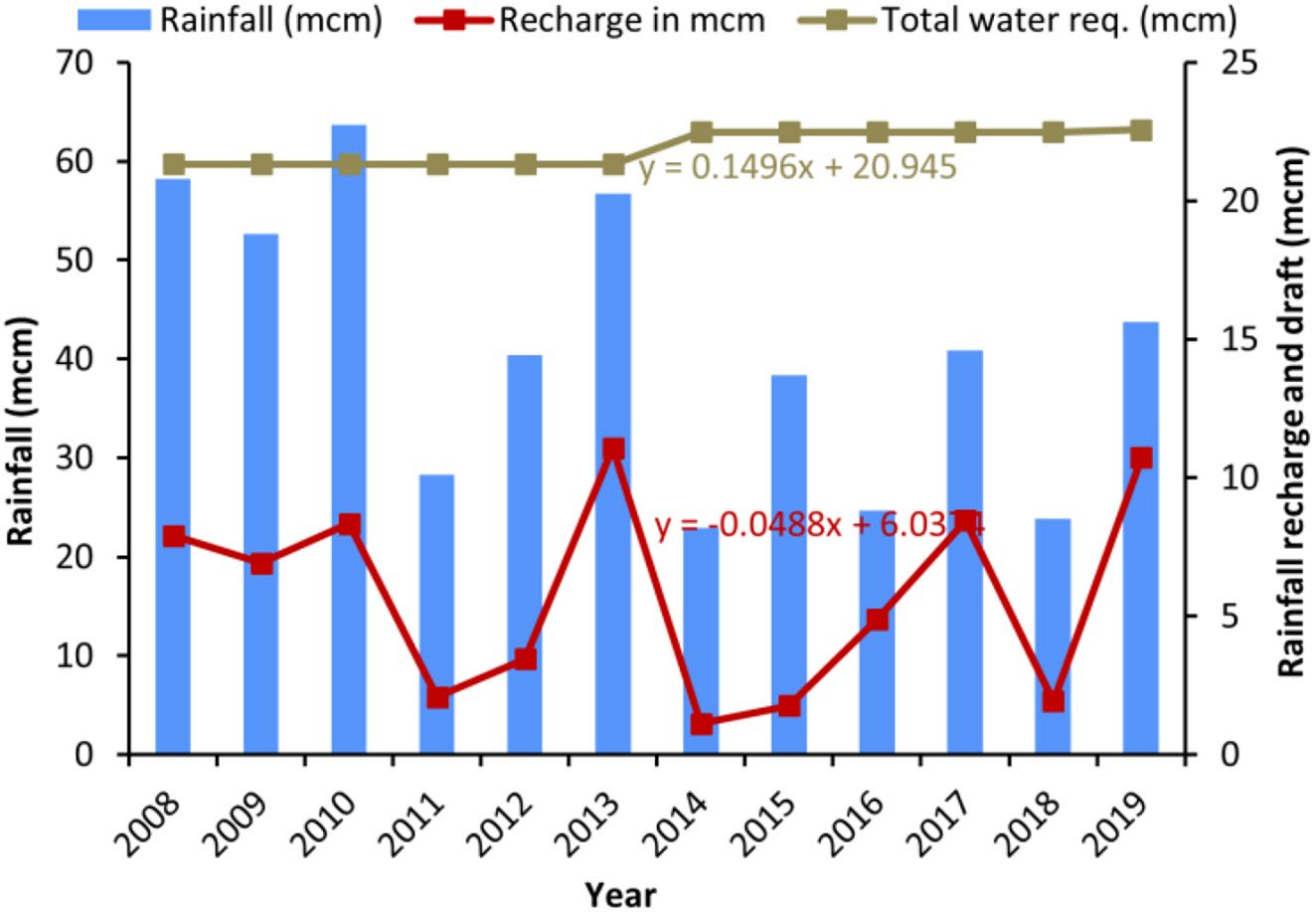
Graph showing the temporal variation of rainfall recharge and draft with respect to rainfall in the study area.

The total estimated dynamic renewable groundwater recharge of the study area varies widely with an average of 5.7 mcm from the last twelve years, while the groundwater draft is 21.9 mcm and, hence the net deficit is 16.2 mcm (Table 3). Figure 7 indicates an increase in groundwater draft from 21.3 mcm in 2008 to 22.6 mcm in 2019 while an impressive rise in groundwater recharge from 7.9 mcm in 2008 to 10.7 mcm in 2019, which is still much lesser than the draft. However, the net recharge in the year 2019 is much higher (10.7 mcm) than the previous year, which is 1.9 mcm. The higher recharge may be attributed to high groundwater pumping during pre-monsoon season in the previous year due to lack of sufficient rainfall. Table 3 shows that the net draft varies from 21.3 mcm to 22.6 mcm and the major groundwater recharge component driving by amount of rainfall and pre-monsoon pumping. On average, 13.1% of precipitation reaches into the groundwater by direct rainfall infiltration and different water storage systems, such as tanks, etc. The percentage of rainfall that participated in the recharge has been significantly improved throughout the years. The total draft indicates the use of massive groundwater for irrigation. Temporal groundwater deficit has been varied throughout the years, and these variations were due to the difference in local hydrological heterogeneity that are variations in rainfall, cropping pattern, irrigational use and domestic uses, etc.

**Table 3:** Groundwater recharge and use in the study area.

Year	Average annual rainfall (mm)	Rainfall (mcm)	Total water use (mcm)	Recharge (mcm)	Deficit (mcm)	Percentage of rainfall participated in recharge
2008	1102	58.2	21.3	7.9	13.4	13.6
2009	997	52.7	21.3	6.9	14.4	13.1
2010	1206	63.7	21.3	8.3	13.0	13.1
2011	535	28.3	21.3	2.1	19.3	7.3
2012	765	40.4	21.3	3.5	17.9	8.5
2013	1074	56.7	21.3	11.1	10.3	19.5
2014	434	22.9	22.5	1.1	21.4	4.9
2015	726	38.3	22.5	1.8	20.7	4.6
2016	467	24.7	22.5	4.9	17.6	19.8
2017	774	40.9	22.5	8.5	14.0	20.7
2018	452	23.9	22.5	1.9	20.6	8.1
2019	828	43.7	22.6	10.7	11.9	24.5
Average	780	41.2	21.9	5.7	16.2	13.1

*mcm* million cubic meters.

The present study suggested that the GIS-based methods of delineating groundwater recharge zones adopted here are valuable tools that can be applied in the different geo-environmental settings. This study also explained the need for local or site-specific soil infiltration information that might enhance the potential recharge zone mapping and help better planning and management of groundwater resources.

## Conclusion

The present research is an attempt to estimate the reliable groundwater recharge potentials by Weighted Overly Analysis using GIS methods using site specific infiltration rates in a semi-arid watershed located in Telangana state, South India. The potential recharge zones have been categorized into four distinct groundwater recharge zones that are good, moderate, poor and very poor. The result shows that good potential recharge areas are mainly found in the valley and low drainage density areas, covering a very small area of about 3.4% considering soil infiltration rates and basement depths along with other thematic layers. The moderate groundwater recharge zones spread all over the catchment area in both the recharge maps, mainly in the shallow weathered pediplains of biotite granite and biotite granite with pegmatite and dyke and low lineament density. Poor and very poor groundwater recharge zones occur predominantly in uplands to lowlands, but very poor recharge zones are relatively lesser in the midlands. The poor and very poor groundwater recharge zones found in the rocky outcrop, high drainage density, steep slope, less lineament density, less or no fissures and barren land regions and covers an area of about 41.4% and 2.2% respectively for the first map. The demarcated potential recharge zone has a good correlation with pre and post-monsoon water level fluctuations.

The groundwater recharge map produced in this study provides valuable information for sustainable land use and groundwater management to enhance agricultural productivity in the region. However, as the study shows a marked decline in precipitation and groundwater levels, it is necessary to consider and understand climatic inconstancy over the long term to plan and manage groundwater resources. The major limitation of the present study is, water budget has been estimated with limited aquifer parameters and distributed rainfall that may influence the total budget and its distribution. The water budget needs to be compared with groundwater modeling results.

To meet the UN Sustainable Development Goals, it is imperative to quantify the inflow and outflow fluxes to the system and, based on their balance, future demand can be managed. However, it is comparatively easy to estimate the outflow fluxes but cumbersome to estimate the inflow fluxes precisely. This study contributes significantly towards this to overcome the limitation of reliable estimation of inflow fluxes.

## Supplementary Information

Supplementary Information.
