# Supplementary material for: Distributed groundwater recharge potentials assessment based on GIS model and its dynamics in the crystalline rocks of South India
Source: Sci Rep. 2021 Jun 3;11:11772. doi: 10.1038/s41598-021-90898-w (PMC8175507; doi:10.1038/s41598-021-90898-w)
Supplement: Supplementary file 1 — Supplementary Information. [file 41598_2021_90898_MOESM1_ESM.docx]

**Distributed groundwater recharge potentials assessment based on GIS model and its dynamics in the crystalline rocks of South India**

**Fauzia Fauzia ^a,b^, L. Surinaidu ^a^, Abdur Rahman ^a,b^ and Shakeel Ahmed ^a^**

^a^CSIR-National Geophysical Research Institute, Hyderabad- 500007, India

^b^[Academy of Scientific & Innovative Research](http://acsir.res.in/) (AcSIR), Ghaziabad- 201002, India

Corresponding author: fauzia@ngri.res.in

Table S1. Details of Infiltration tests.

| Test No. | Latitude | Longitude | Infiltration Rate (mm/hr) |
| --- | --- | --- | --- |
| 1 | 17.170738 | 78.461675 | 37.37 |
| 2 | 17.137839 | 78.431931 | 28.03 |
| 3 | 17.151747 | 78.457117 | 13.87 |
| 4 | 17.15817 | 78.42902 | 25.48 |
| 5 | 17.13035 | 78.44471 | 60.86 |
| 6 | 17.13567 | 78.45709 | 59.87 |
| 7 | 17.15441 | 78.41178 | 22.93 |
| 8 | 17.14467 | 78.42245 | 8.92 |
| 9 | 17.12879 | 78.41421 | 80.68 |
| 10 | 17.10746 | 78.42942 | 132.48 |
| 11 | 17.12495 | 78.43797 | 64.12 |
| 12 | 17.16072 | 78.47348 | 48.83 |
| 13 | 17.15405 | 78.44198 | 46.71 |
| 14 | 17.12464 | 78.46181 | 55.20 |
| 15 | 17.18573 | 78.46744 | 91.30 |


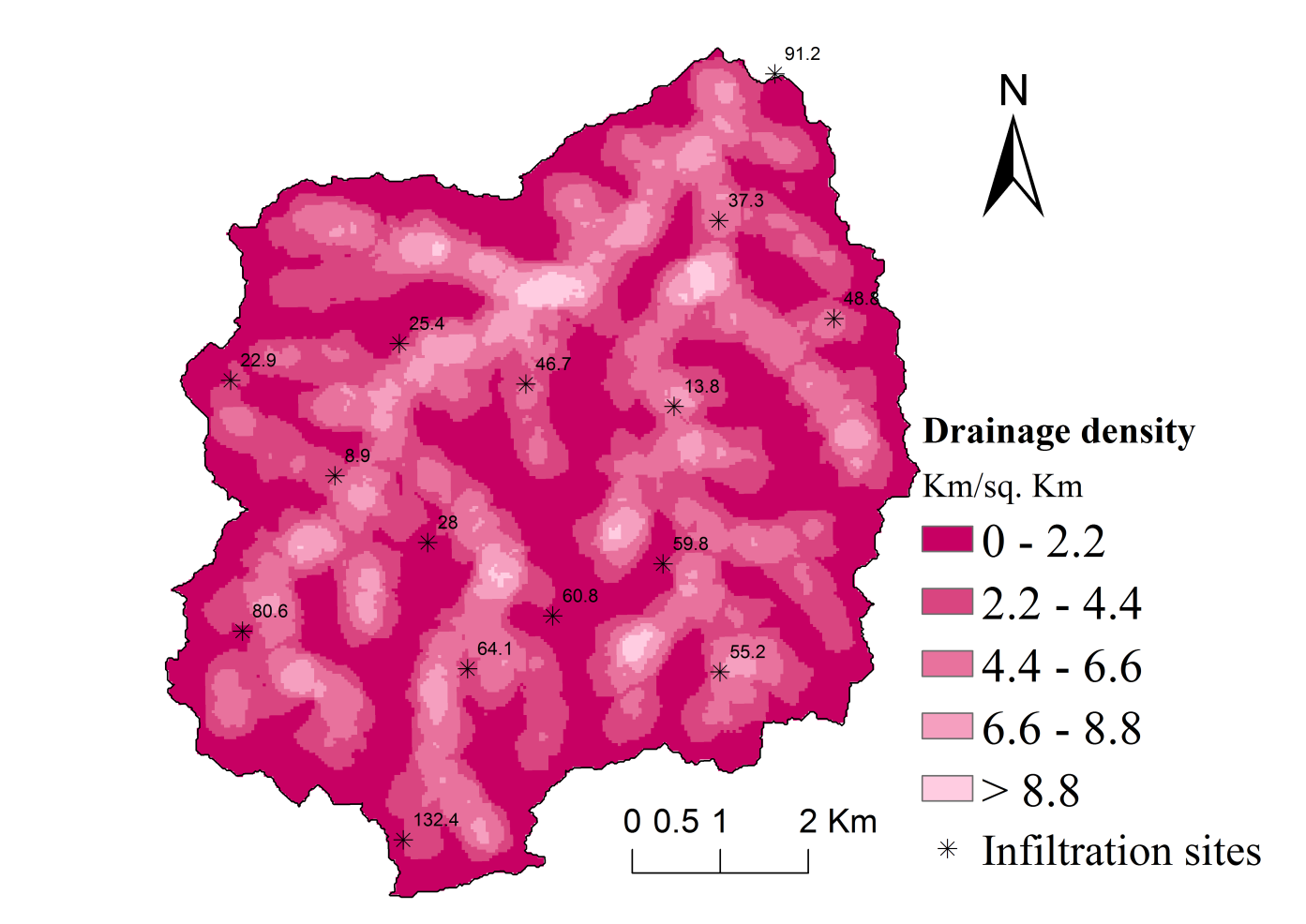


Figure S1. Drainage density with infiltration sites and values.


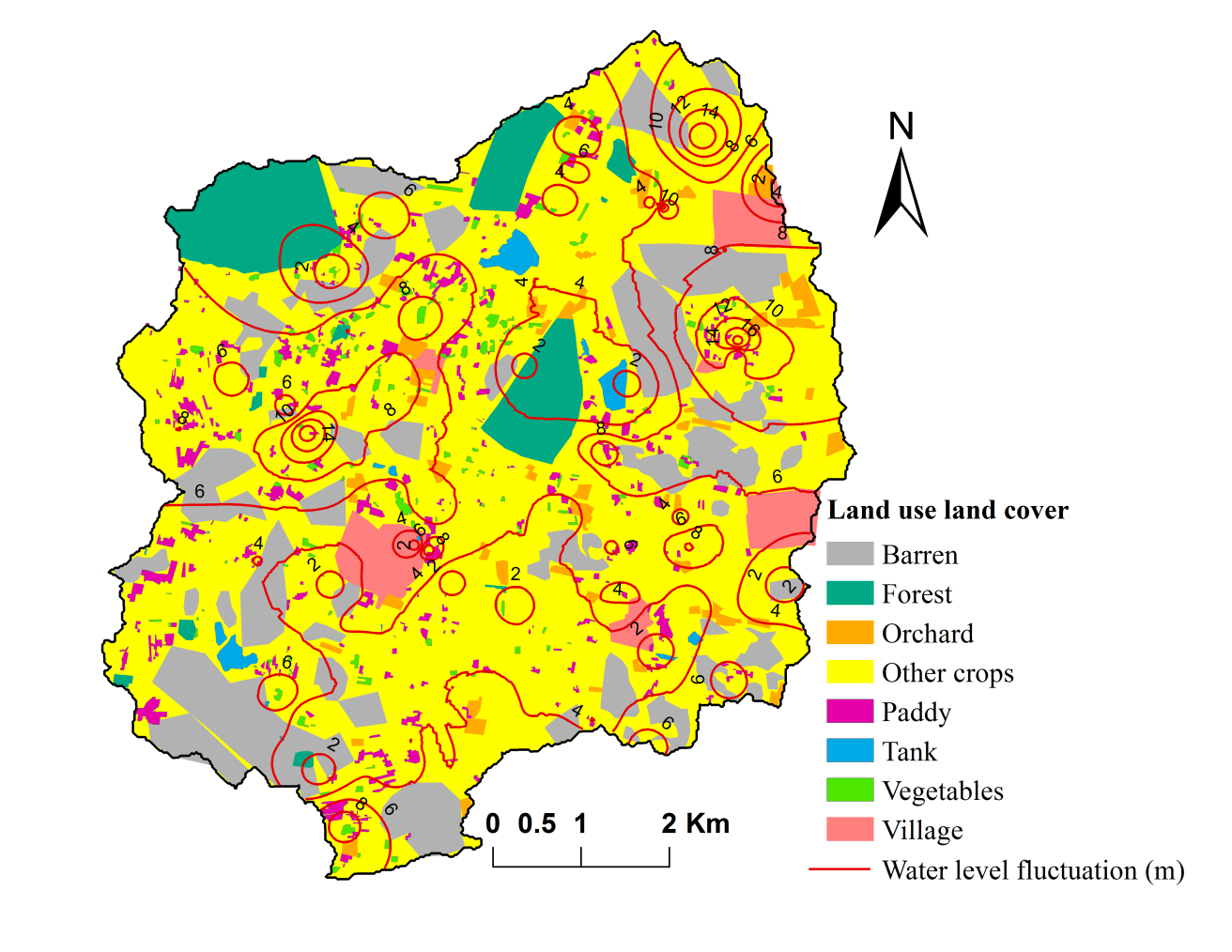


Figure S2. LULC with water level fluctuation
